# Epithelial-to-mesenchymal plasticity of cancer stem cells: therapeutic targets in hepatocellular carcinoma

**DOI:** 10.1186/s13045-016-0307-9

**Published:** 2016-08-30

**Authors:** Aparna Jayachandran, Bijay Dhungel, Jason C. Steel

**Affiliations:** The University of Queensland School of Medicine and the Gallipoli Medical Research Institute, Greenslopes Private Hospital, Brisbane, Queensland Australia

**Keywords:** Hepatocellular carcinoma, Cancer stem cells, Cancer-initiating cells, Epithelial-to-mesenchymal transition, Cellular plasticity, Tumor heterogeneity, Drug resistance

## Abstract

Hepatocellular carcinoma (HCC) remains one of the most common and lethal malignancies worldwide despite the development of various therapeutic strategies. A better understanding of the mechanisms responsible for HCC initiation and progression is essential for the development of more effective therapies. The cancer stem cell (CSC) model has provided new insights into the development and progression of HCC. CSCs are specialized tumor cells that are capable of self-renewal and have long-term repopulation potential. As they are important mediators of tumor proliferation, invasion, metastasis, therapy resistance, and cancer relapse, the selective targeting of this crucial population of cells has the potential to improve HCC patient outcomes and survival. In recent years, the role of epithelial-to-mesenchymal transition (EMT) in the advancement of HCC has gained increasing attention. This multi-step reprograming process resulting in a phenotype switch from an epithelial to a mesenchymal cellular state has been closely associated with the acquisition of stem cell-like attributes in tumors. Moreover, CSC mediates tumor metastasis by maintaining plasticity to transition between epithelial or mesenchymal states. Therefore, understanding the molecular mechanisms of the reprograming switches that determine the progression through EMT and generation of CSC is essential for developing clinically relevant drug targets. This review provides an overview of the proposed roles of CSC in HCC and discusses recent results supporting the emerging role of EMT in facilitating hepatic CSC plasticity. In particular, we discuss how these important new insights may facilitate rational development of combining CSC- and EMT-targeted therapies in the future.

## Background

Hepatocellular carcinoma (HCC) is the most commonly diagnosed malignancy of the liver and is the third most frequent cause of cancer mortality worldwide [1–4]. HCCs are highly aggressive carcinomas that are often fatal due to high level of tumor invasiveness, intrahepatic spread, and extrahepatic metastasis [5, 6]. HCCs are multifactorial and its incidence is highly correlated to chronic inflammation and cirrhosis. Chronic hepatitis B and C infections and alcohol overconsumption are considered to be risk factors for HCC [7–9]. The prognosis for patients with advanced HCC remains extremely poor due to the high rates of recurrence and metastasis. Conventional treatments for HCC patients such as liver resection, transplantation, and chemotherapy have shown limited efficiency in advanced disease [10–12]. Thus, the ultimate goal in combating HCC in advanced stages is to overcome therapeutic resistance and to prevent disease recurrence.

**Fig. 1:**
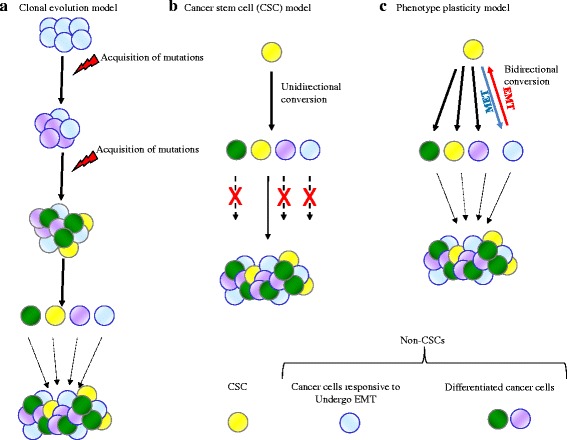
Different models of tumor heterogeneity. **a** Clonal evolution or stochastic model suggests that serial acquisition of mutations generates tumor cell heterogeneity and all cells are capable of renewal and tumorigenesis. **b** According to the cancer stem cell (CSC) model, tumors are organized into a hierarchy of heterogeneous cell populations, and only a small subset of cells within a tumor called CSCs have the ability to sustain tumor formation. CSCs have the ability to perpetuate themselves through self-renewal and generate large populations of more differentiated descendants by unidirectional conversion. **c** Phenotype plasticity model posits that irreversibly differentiated cells can be converted back to an undifferentiated state or stem cell-like state given the appropriate stimulus. This dynamic bidirectional conversion between CSC and non-CSC can give rise to tumor heterogeneity

The precise molecular mechanisms of HCC pathogenesis are unclear. HCC features significant genetic, phenotypic, and functional heterogeneity, with the potential to confound the success of many therapies. A molecular basis of heterogeneity in HCC was evidenced by studies that found markedly different molecular profiles among cells from clinical specimens [13–15]. HCC intratumoral heterogeneity is a hallmark feature that represents a substantial obstacle to achieving favorable clinical response in patients. Clonal evolution, cancer stem cell, and phenotype plasticity models have been postulated to explain how tumor cell heterogeneity arises (Fig. 1). These models are essentially used to describe cancer development, with the differences between the models having important implications for the rational design of drugs and treatment strategies.

Clonal evolution or stochastic model suggests that serial acquisition of mutations generates tumor cell heterogeneity and contributes to cancer progression. With each new advantageous mutation, a clonal growth of novel cell populations completely or partially overgrows the old [16, 17]. In accordance with this model, most cancer cells possess the mutations and molecular changes that gave the cells their malignant properties, and therefore, removing the bulk of the tumor will curtail tumor progression. However, the view that every cancer cell has the same or equal potential to support disease progression has long been challenged. In the early 1970s, it was recognized that not all cancer cells are capable of extensive proliferation in colony formation assays [18]. This has been expanded to in vivo studies showing that not all cells within a cancer are able to initiate tumors when implanted into mice [19].

The second model of cancer stem cell (CSC) or cancer-initiating cell (CIC) theory supports the presence of a specific subpopulation of cancer cells that possess tumorigenic potential and generates tumor cell heterogeneity [20]. According to this model, the identification of targeted therapies to remove the CSCs would lead to tumor regression and disease stabilization. This theory postulates the existence of a unidirectional differentiation hierarchy, where non-CSCs cannot generate CSCs. However, it is becoming increasingly apparent that differentiated cells can be switched to generate CSCs [21].

Phenotype plasticity model posits that irreversibly differentiated cells can be converted back to an undifferentiated state or stem cell-like state given the appropriate stimulus [22, 23]. This model suggests that CSCs are a dynamic subpopulation of cancer cells rather than a stable cell population and has important implications for the design of combination therapies. Several intriguing studies have described that HCC cells hijack portions of the developmental epithelial-to-mesenchymal transition (EMT) program to generate CSCs, thereby facilitating metastasis and drug resistance [24–26]. EMT process provides a means to link clonal evolution and CSC models and forms the basis for phenotypic plasticity model [21]. Here, we summarize new insights into the molecular mechanisms that link CSCs and EMT in HCC. Deciphering the relationships between these fundamental processes will expand our knowledge of the underlying etiology and pathogenesis of HCC and lead to development of novel clinical targets and improve the clinical management of HCC patients.

## Cancer stem cells

According to the CSC hypothesis, tumors are organized into a hierarchy of heterogeneous cell populations, and only a small subset of cells within a tumor, termed CSCs or CICs, have the ability to sustain tumor formation and growth [27–29]. The CSC model has provided an important conceptual framework that has proven highly useful for understanding intratumoral heterogeneity [30]. CSCs are similar to normal stem cells in their ability to perpetuate themselves through self-renewal and generate large populations of more differentiated descendants [27, 31]. The differentiation of CSCs results in the recapitulation of the cellular heterogeneity of the original tumor. CSCs can seed tumors when transplanted into immune-compromised animal host [19, 20, 32]. CSCs may also exhibit inherent drug resistance and enhanced invasive and migratory potential that implicate a role in disease pathogenesis spanning initial tumor formation to metastatic disease progression [33]. The CSC model also provides a framework for therapeutic failure and relapse [34]. These hurdles can be overcome by characterization of CSCs and the identification of specific targeted therapies to eliminate CSCs. Given that CSCs exist in most hematological and solid tumors, they have emerged as potential targets [28, 29, 35, 36]. CSCs have been identified and harvested using a number of different strategies that have been extensively reviewed elsewhere [37, 38].

## Cancer stem cells and their implications in HCC

CSCs have proven to play a central role in the development, maintenance, metastasis, and recurrence of HCC [39–42]. Initially, CSC populations were identified and isolated from human HCC cell lines and xenograft tumors characterized by their expression of CD133, a cell surface glycoprotein [43, 44]. CD133^+^ cancer cells exhibited stem cell-like properties, including higher proliferative potential, greater colony-forming efficiency, self-renewal, and differentiating capacity when compared to CD133^–^ counterparts. Additionally, CD133^+^ cells could initiate tumor growth in vivo, suggesting validation of the CD133 marker for enrichment of CSCs in HCC [43]. Besides CD133 expression, HCC cells possessing CSC attributes have been reported to express diverse cell surface CSC markers such as CD90, CD44, CD13, and epithelial cell adhesion molecule (EpCAM) [39, 45–48]. Isolating CSCs on the basis of single markers has, however, proven to be not definitive, with cells derived from negative populations often inducing tumors albeit at a reduced capacity. Hence, utilizing a combination of different markers that co-express would be a better strategy to isolate CSCs from HCC. For instance, co-expression of surface markers including CD44, CD90, and CD133 has been used to isolate CSCs in HCC [49]. However, co-expression of CSC markers has shown the existence of heterogeneity within the CSC populations of even the same patient tumor samples [50]. A recent study has demonstrated that heterogeneous CSC populations interact and influence functional traits within a single tumor. Notably, upon co-culturing CD90^+^ and EpCAM^+^ CSC populations, CD90^+^ CSCs were able to promote motility in non-motile epithelial-like EpCAM^+^ CSCs [45].

Although a number of molecules have been identified as markers for CSC, there is no general consensus on the best CSC markers for HCC. Moreover, there is high variability in marker expression across established cell lines and HCC tumors and their suitability for therapeutic targeting has not been extensively evaluated [49, 51]. Hence, there is still a critical need for identification of more robust markers for the enrichment of hepatic CSC. Side population flow cytometry using Hoechst 33342 staining and sphere formation assays based on functional aspects of CSCs have been utilized for enriching hepatic CSCs [52, 53]. Tumorspheres possess the capacity for self-renewal and tumorigenicity and thus represent a more precise tool for the enrichment of CSCs [54]. For a comprehensive review on methods to isolate CSCs in HCC, see Chiba et al. [55].

## EMT: an essential developmental process reactivated during cancer progression

EMT is a complex molecular and cellular reprograming process by which epithelial cells lose their differentiated characteristics and acquire mesenchymal features, including motility, invasiveness, ability to escape immune cells, and a heightened resistance to apoptosis [56]. EMT is an essential process for morphogenetic events in embryonic development which enables immobile epithelial cells to gain a motile mesenchymal phenotype [57, 58]. In cancers, the inappropriate induction of this developmental process can be disastrous as they endow differentiated sessile epithelial cells with migratory and invasive traits thereby initiating the first step of tumor metastasis cascade [57, 59]. In support of this idea, EMT has been documented at the invasive fronts of several cancer types [60, 61]. Mesenchymal cells can undergo a reverse phenotype switching to regain epithelial state via mesenchymal-to-epithelial transition (MET) to colonize new sites and create secondary tumors in distant organs [62]. Thus, EMT is a dynamic and reversible process, which allows cancer cells to reversibly transition between epithelial and mesenchymal states during the metastasis process. Phenotypic switching of cells influences the prognosis of cancer patients by promoting multidrug resistance of tumors and recurrence [62, 63]. The molecular events driving EMT have been delineated and reviewed in detail by Lamouille et al. [64].

## EMT phenotypic cells as a resource for hepatic CSCs

Recently, EMT has emerged as an important regulator of cancer cells exhibiting stem cell-like properties [65]. The best established example of the generation of CSC from their non-CSC progeny comes from recent studies relating breast CSC and EMT [62, 66]. These studies demonstrated that the induction of EMT in immortalized human mammary epithelial cells lead to the expression of both mesenchymal markers and CD44^high^CD24^low^ cell surface marker profile characteristic of breast CSCs. Notably, these cell populations also acquired self-renewal properties and enhanced tumor-initiating ability [62, 66]. Similar studies have revealed the co-expression of both EMT- and CSC-associated genes at the invasive front of colorectal cancer and in spindle tumor cells inside the blood vessels of patients with metastasis [63, 67]. Furthermore, compelling evidence for the EMT-CSC link was provided by studies demonstrating the induction of EMT in cancer cells resulted in the expression of CSC markers [62, 66, 68]. These studies reinforced the possibility that CSC and EMT are mechanistically correlated and may be key components of cancer progression and metastasis.

Transcription factors Snail and Slug have been established as crucial regulators of EMT during embryonic development, organ fibrosis, and cancer progression, as they are potent repressors of E-cadherin expression. Overexpression of Slug but not Snail has been reported in the HCC cell line, HepG2, to induce EMT and enhance CSC marker CD133 [69]. The EMT-CSC link may also be exploited to enrich for CSCs. Mitra et al. identified the existence of Vimentin on the surface of liver CSCs and utilized this in a separation technique to enrich EMT-positive CSCs directly from primary tumor cells [70]. Similarly, Li et al. enriched CSCs from human HCC cell lines and human primary HCC tumors by the sphere-forming assay. These cells exhibited high expression of CD90 and mesenchymal marker Vimentin [71].

The connection between EMT and CSC indicates that the EMT process is doubly dangerous for the cancer patient and may explain how CSCs maintain their aggressiveness in the long period of time when invading and migrating to surrounding tissues [65]. Notably, the link between CSC and EMT markers has been explored in HCC patient cohort. EMT and CSC marker expression examination in 27 HCC patients by immunohistochemistry revealed downregulation of epithelial marker E-cadherin in 63 % of patients and upregulation of mesenchymal marker N-cadherin in 81 % of patients. This patient cohort also showed upregulation of CSC marker CD13 in 78 % of patients [72]. Given that both EMT and CSC phenotypes are crucial for tumor progression and metastasis, an in-depth investigation of crosstalk of CSCs with EMT has important implications for our understanding of HCC progression.

## EMT: the driver of hepatic CSC plasticity

EMT may provide a means to integrate the canonical CSC model with the clonal evolution model. Given that EMT drives a dedifferentiation-like process whereby non-CSCs can be reprogramed to gain a CSC phenotype, it supports a bidirectional CSC model. Chaffer et al. proposed that some tumors adhere to phenotypic plasticity model wherein dynamic bidirectional conversions between the CSC and non-CSC compartments are common and essential components of tumorigenicity. A study on breast cancer suggests that aggressive CSCs can be created de novo within a tumor by spontaneous mechanisms that pertains to a switch in cell phenotype and is not driven by additional genetic mutations [21]. Another study revealed that more differentiated breast cancer cells can give rise to cells expressing breast CSC markers and that the rate of interconversion is variable between cells from different lesions [73]. CSCs might be generated with processes that are related to activation of EMT, which impacts cell differentiation and tumor metastatic potential [74]. Phenotypic plasticity would provide an extremely robust system as most or all cells could regenerate heterogeneity if a particular cell type was removed, in contrast to the reliance on random mutations implicit in clonal evolution.

Recent data suggests that CSCs are indeed plastic and can be associated with both epithelial and mesenchymal states. Occurrence of two distinct populations of CSCs termed EMT-CSCs with epithelial signature and MET-CSCs with mesenchymal signature have been reported in breast cancer. These CSCs have the capacity to transition between these cellular states. It has been envisioned that EMT-CSCs are located at the invasive edge of the tumor and allow the tumor to expand into new territory. On the other hand, MET-CSCs are located in the tumor interior and facilitate tumor cell growth. Furthermore, when the invasive edge becomes the interior of the tumor, the two CSCs can change cellular states [75]. It is conceivable that CSCs with distinct EMT/MET phenotypes also occur in HCC. In support for this hypothesis, CSCs with mesenchymal phenotypes were observed in the invasive front of HCC patient samples [24, 76]. In an earlier study of CSC populations defined by CD133^+^/ALDH^high^ and CD133^+^/EpCAM^+^ separated from Huh7, HepG2, and Hep3B, well-differentiated human HCC cell lines were deemed to be epithelial by expression of E-cadherin and lack of Vimentin. In contrast, poorly differentiated cell lines such as SkHep1, HLE, and HLF and double negative subpopulation from well-differentiated cell lines were characterized as mesenchymal due to the higher expression of Vimentin, Zeb1, and Snail; and transforming growth factor (TGF)-β and hedgehog pathway activation [77]. Another study examined microarray data from 238 HCC cases and found several distinct subpopulations characterized by expression of CD133, CD90, and EpCAM. Gene expression revealed an enriched EMT signature in CD90^+^ cells. Further examination of both primary tumors and HCC cell lines demonstrated that CD90^+^ subpopulation of cells were mesenchymal and overexpressed CD44, c-KIT, and TWIST1, whereas EPCAM^+^ subpopulation were epithelial and expressed alpha-fetoprotein (AFP) and Albumin [45]. The plasticity of these distinct subpopulations to switch between different EMT cellular states warrants further investigation.

## EMT phenotype is linked with the biology of CSC in HCC

Recent studies in HCC have demonstrated that the EMT phenotype and CSC biology are intricately linked. Thus, identifying common molecular pathways regulating CSC and EMT attributes may be critical for the development of efficacious treatment strategies for HCC patients. Table 1 provides a list of cell surface molecules, transcription factors, microenvironmental cues, and micro RNAs (miRNAs) that have been implicated in the molecular pathways linking CSCs to EMT in HCC. Recent studies provide evidence for the emergence of cells with combined EMT and CSC phenotypes. For example, cell surface marker Keratin19^+^ (K19^+^) expressing human HCC cells exhibited CSC-like properties together with elevated EMT marker expression. Activation of TGF-β and Smad signaling axis in K19^+^ cells has been implicated in mediating EMT [24]. Similarly, another study revealed that CD44, a putative CSC marker, is associated with a mesenchymal phenotype in HCC cell lines. CD44^+^ cells expressed high levels of mesenchymal markers N-cadherin and Vimentin and low levels of epithelial marker E-cadherin. Knockdown of CD44 reversed EMT by repression of ERK/Snail pathway and inhibited lung metastasis of HCC cells in the metastatic model of HCC established by tail vein injection of luciferase-labeled HCC cells in nude mice [78]. A recent study found that CD44 protein levels were enhanced by TGF-β1 treatment and that synergistic interactions between CD44 and TGF-β1 induced EMT and CSC phenotypes through Akt/GSK-3β/β-catenin signaling axis in HCC [79]. Another study demonstrated that by targeting CSC marker CD133, CSC and EMT traits can be abrogated [26]. These studies have identified a clear functional link between EMT and CSC, as altered expression of CSC cell surface markers can conversely affect the EMT process.

**Table 1:** Determinants of CSC and EMT in HCC

Molecular regulator	Signaling pathway	Study
Cell surface markers		
K19^+^	TGF-β-Smad	Kawai et al. [24]
CD44^+^	ERK/Snail;	Gao et al. [78]
	TGF-β/Smad; Akt/GSK-3β/β-catenin	Park et al. [79]
CD133^+^	NF-kB	Liu et al. [26]
CD90^+^		Yamashita et al. [45]
EpCAM		Yamashita et al. [45]
Vimentin^+^CD133^-^		Mitra et al. [70]
Transcription factors		
Nanog	Stat3/Snail	Yin et al. [25]; Yu et al. [80]
Oct4	Stat3/Snail	Yin et al. [25]
Slug		Sun et al. [69]
Sox9	TGF-β/Wnt/β-catenin	Kawai et al. [82]
Oncogene		
Malestrom	Akt/GSK-3β/Snail	Liu et al. [81]
Microenvironmental cues		
Hepatic stellate cells	HGF/Met	Yu et al. [91]
Tumor-associated macrophages	TGF-β1	Fan et al. [76]
Artemin		Zhang et al. [83]
Hypoxia	Akt	Zhang et al. [83]
	Twist1/Bmi1	Liu et al. [92]
	HIF-1α/Artemin/Akt	Zhang et al. [83]
Oxidative stress		
HCV infection	Ca^2+^ signaling	Iqbal et al. [94]
Folate deficiency	miR-22	Su et al. [95]
Viral infection		
HCV geneotype 2A	Hypoxia/osteopontin/Akt/GSK-3β/β-catenin	Kwon et al. [84]
		Iqbal et al. [94]
miRNA		
miR-200a	Wnt/β-catenin	Liu et al. [87]
miR-125b	TGF-β/SMAD2/4	Zhou et al. [72]
miR-148b		Liu et al. [88]
miR-200b, miR-200c, miR-122, miR-145	DDX3	Li et al. [89]
Sphere formation	Notch	Li et al. [71]

Pluripotency factors, oncogenes, and viral infection that result in the generation of CSCs in HCC, also confer these cells with EMT features. For instance, ectopic expression of pluripotency associated transcription factors Oct4 and Nanog that are essential for maintenance of stem cell phenotype endowed HCC cell line MHCC97-L with both CSC and EMT traits through the activation of signal transducer and activator of transcription 3 (Stat3)/Snail signaling [25]. Furthermore, Yu et al. demonstrated that disruption of Nanog expression resulted in downregulation of pluripotency factors Oct4, Klf4, and Sox2; and CSC marker CD133 and reversal of EMT [80]. Another study examined the effect of oncogene Maelstrom that is generally silenced at a transcriptional level in somatic tissues, on the acquisition of CSC and EMT features in HCC cells. Liu et al. demonstrated that Maelstrom upregulated stemness-associated genes (Nanog, Oct4, Bmi-1, Notch-1, and Smo), CSC markers (CD24, CD44, CD133, CD105, and CD166) and EMT markers (Snail, Slug, Vimentin, and Fibronectin). They also demonstrated that Maelstrom ectopic expression in HCC cells is associated with the acquisition of EMT and CSC features through the activation of AKT/GSK-3β/Snail axis [81]. Similarly, sex-determining region Y-box 9 (SOX9), a transcription factor expressed in embryonic liver but not in adult hepatocytes, has been found to induce EMT and CSC traits via the activation of TGF-β/Smad and Wnt/β-catenin signaling pathways [82].

Similarly, Artemin, an estrogen-regulated growth factor which promotes resistance to antiestrogen therapies, has been shown to modulate EMT features of HCC cells with downregulation of E-cadherin and upregulation of N-cadherin, Vimentin, Snail, and E47. Artemin promotes the metastatic properties and tumor-initiating capacity of HCC by AKT modulation of factors involved in EMT and stemness [83]. Chronic hepatitis C virus (HCV) infection, an important factor in the etiology of HCC, is also associated with EMT and CSC pathways. Human primary hepatocytes infected with cell culture grown HCV genotype 2a revealed enhanced EMT markers (Snail, Slug, Twist, and Vimentin) and stemness markers (CD133, Nanog, Lin28A, Oct4A, Notch-1, c-Myc, and c-Kit) [84].

Recent studies of non-coding RNAs are shedding light on the regulation of EMT and CSC in cancers [85, 86]. Studies focused on hepatic oval cells as the source of hepatic CSCs have uncovered miR-200a as a suppressor of EMT and CSC signatures. Stable knockdown of miR-200a conferred mesenchymal and CSC characteristics to rat hepatic oval cell line WB-F344 via activation of Wnt/β-catenin pathway [87]. Additionally, in a cell culture model where HCC cell lines were treated with 10 ng/ml TGF-β1 for 4 to 6 days, miR-125b was found to be downregulated. miR-125b blocked EMT and exerted its inhibitory effects via SMAD2 and 4 [72]. Another study revealed that miR-148b was downregulated in HCC side populations (SP) that were selected based on efflux of Hoechts 33342 dye. SP cells of HCC were enriched for CSC-like properties. Functional studies demonstrated that miR-148b abrogated the expression of CSC and EMT markers [88]. Recently, reduced expression of DDX3, a member of the DEAD-box RNA helicase family, has been correlated with poor HCC patient survival and conferred HCC cells with CSC and EMT traits. Moreover, DDX3 expression positively correlated with transcription of several tumor-suppressive miRNAs, namely, miR-200b, miR-200c, miR-122, and miR-145 [89].

In addition to intracellular factors influencing EMT and CSCs, recent studies have found that interaction among cancer cells and stromal cells within the tumor microenvironment can induce EMT by secretion of mediators such as growth factors, cytokines, and extracellular matrix proteins [90]. Accordingly, reciprocal signaling between hepatic stellate cells and precancerous hepatoma cells induced an EMT and CSC-like properties in HCC mediated by hepatocyte growth factor (HGF)/Met signaling [91]. Similarly, exposure to conditioned media from tumor infiltrating macrophages (TAMs) rich in TGF-β1 promoted hepatoma cells to undergo EMT and to gain CSC-like properties [76]. These studies reinforce the involvement of tumor stroma in mediating EMT and generating CSC in HCC.

Rapid growth of cancer cells often creates insufficient supply of oxygen and results in hypoxic microenvironment. A hypoxic microenvironment is a common feature of HCC and is associated with malignant invasion, metastasis, EMT, and CSC [92, 93]. A previous study has established that hypoxia upregulated the expression of Twist1 and Bmi1, two proteins that have important roles in inducing EMT and cancer cell stemness [92]. Another study has demonstrated that HCV infection of human HCC cells induced osteopontin that triggered an EMT process via the Akt/GSK-3β/β-catenin axis [94]. Furthermore, this study demonstrated that osteopontin induces downstream signaling cascade via the receptor CD44, a marker of CSC in HCC. Moreover, a concomitant elevation of ROS in the mitochondria of HCV infected HCC cells as a result of enhanced Ca^2+^ signaling has implicated the importance of oxidative stress in promoting EMT and CSC in HCC [94]. A recent study demonstrated that HCC cells cultured under folate deficiency culture condition elicited a significant increase in intracellular reactive oxygen species, accompanied by activation of EMT and CSC phenotypes. Mechanistically, this study showed decreased miR-22 level leads to EMT and CSC traits under folate deficiency [95]. These studies have suggested oxidative stress as a key factor in promoting metastasis. However, detailed mechanistic explanations linking oxidative stress to CSC and EMT in HCC remains to be elucidated.

The transcription factor hypoxia-inducible factor-1α (HIF-1α), a key mediator of the cellular response to hypoxia, is overexpressed in HCC [96]. A recent study suggested that during hypoxia, HIF-1α upregulated Artemin expression and this in turn promoted CSC and EMT functions in HCC [83]. Won et al. demonstrated that hypoxia could trigger Stat3-mediated CD133 upregulation with concomitant enhanced HIF-1α levels [96]. Interestingly, another study demonstrated that Frizzled2 (FZD2) activated Stat3 signaling induced EMT in HCC [97]. More studies are needed to better understand the contribution of hypoxia and Stat3 in mediating EMT and CSC in HCC. Collectively, these studies suggest a relationship between tumor hypoxia, EMT, and CSC. However, the molecular mechanisms that relay the hypoxia signal into hepatic EMT and CSC are still largely elusive and warrant further investigation.

## EMT- and CSC-associated resistance to cancer therapeutics

Recent evidences indicate that HCC patients manifesting both CSC and EMT phenotypes are unresponsive to standard chemotherapies and have low progression free survival [24]. This may be explained by the persistence of CSCs, partly generated and maintained by EMT. CSCs are inherently resistant to drugs and toxins through high expression of several ATP-binding cassette (ABC) transporters, an increased DNA repair capacity, and resistance to apoptosis [20, 98]. HCC cells double-positive for CSC and EMT markers were more resistant to chemotherapeutic agents such as cisplatin, doxorubicin, paclitaxel, and sorafenib due to elevated expression of ABC superfamily transporters ABCC2, ABCG2, and MDR1 [25, 81, 87]. Others demonstrated that K19^+^ HCC cells were resistant to 5-Fluorouracil treatment by high expression of a drug transporter, multidrug resistance protein 5 (MRP5) [24]. Sorafenib, an orally-available kinase inhibitor, is the only standard clinical treatment against advanced HCC. Increasing evidence suggests that sorafenib resistance in HCC is correlated with the activation of EMT and enrichment of CSC traits [99–101]. Furthermore, EMT and CSC have been implicated as key mechanisms leading to tumor recurrence in patients who received radiofrequency ablation (RFA) treatment for local control of HCC [102]. Identifying the mechanisms by which EMT-transformed CSCs initiate relapse could facilitate the development of new or enhanced personalized therapeutic regimens.

## Therapies targeting CSC and EMT phenotypes

**Fig. 2:**
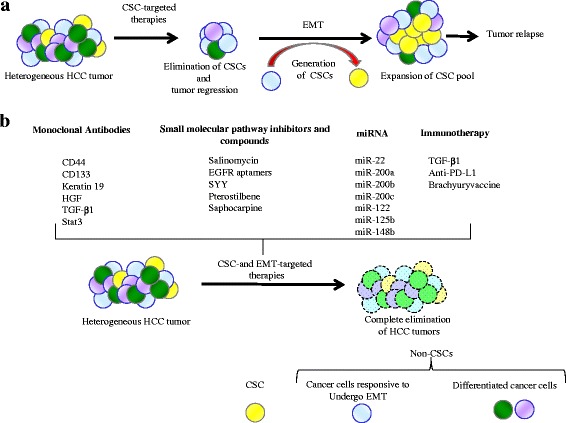
Possible targeted therapies for the treatment of HCC. **a** HCC tumor treatment modalities with CSC mediated treatment initially result in tumor shrinkage and regression due to the elimination of CSCs. Some residual non-CSCs undergo EMT leading to repopulation of CSCs, subsequently resulting in treatment failure and metastasis. **b** CSC and EMT therapies comprising of treatments targeting CSC and EMT phenotypes with monoclonal antibodies, pathway inhibitors, miRNAs, or immunotherapy potentially leads to improved outcomes

The association of EMT and CSCs may also form the basis for identifying novel targeting agents to improve clinical outcomes in HCC patients (Fig. 2). One potential approach is using monoclonal antibodies to target the CSC cell surface antigens that regulate EMT. For example, the use of specific monoclonal antibodies against CD44 or CD133 may prove to be effective in completely eliminating HCC cells with CSCs and EMT phenotypes. As evidence for this, recent studies have demonstrated that shRNA mediated CD44 or CD133 knockdown reversed the EMT phenotype [26, 78]. Another approach is to perform high-throughput chemical screen to identify compounds that could induce cell death in CSCs generated by EMT. The ionophore salinomycin was identified as a potential CSC targeting agent that subsequently blocked tumor formation and metastasis in vivo in different malignancies [103, 104]. Recent studies have revealed that salinomycin was found to exert synergistic cytotoxicity in combination with chemotherapy drugs such as doxorubicin and fluorouracil (5-FU) in HCC cells. These combination treatments were able to target both CSC and EMT phenotypes [105, 106]. Furthermore, salinomycin-loaded poly(lactic-co-glycolic acid) nanoparticles conjugated with both CD133 and epidermal growth factor receptor (EGFR) aptamers could effectively target HCC cells simultaneously expressing EMT mediator EGFR and CD133 [107]. The activation of EGFR is known to elicit an EMT in HCC [108]. Given the success of salinomycin in pre-clinical studies in HCC, it can be envisaged that it will be the object of future clinical studies. Treatment of HCC cell lines with Songyou Yin (SYY, a traditional Chinese medicine containing five herbal compounds) reduced CSC markers (CD90, CD133, CD44, CD24, and EpCAM) and mesenchymal marker Vimentin and restored epithelial marker E-cadherin. Furthermore, combination treatment of SYY and Oxaliplatin in nude mice bearing orthotopic xenografts reduced both CSC and EMT phenotypes [109, 110]. Pterostilbene, a compound isolated from red sandalwood and blueberry reduced CD133 subpopulation of human HCC cell line Mahlavu. Pterostilbene treatment also reversed EMT in these cells by increasing the expression of E-cadherin and suppressing the expression of mesenchymal markers Vimentin and Twist 1 [111]. Recently, treatment with Sophocarpine, a compound derived from the foxtail-like sophoraherb yielded fewer CSCs in HCC cell lines HCC-LM3 and MHCC-97H. Sophocarpine treatment reduced stem cell markers and inhibited TGF-β-induced EMT [112].

As EMT signaling is involved in development and maintenance of hepatic CSCs, an improved understanding of EMT signaling networks and identifying key molecular players linking these cellular phenotypes may uncover new therapeutic targets. For instance, HGF/MET signaling axis is a potential target as HGF neutralizing antibody effectively reduced activation of HGF/MET signaling and concurrently blocked promotion of EMT and CSC phenotypes and cisplatin resistance [91]. Inhibition of TGF-β1 pathway with a neutralizing antibody or a TGF-β receptor 1 inhibitor has shown to block EMT and CSC traits in HCC cell line [24, 76]. Interestingly, dual inhibition of CD44 and TGF-β1 exerted an anti-metastatic effect with reduced migration and sphere formation more strongly than inhibition of either one alone [79]. Targeting Stat3 signaling pathway may represent another potential approach to overcome EMT process in HCC displaying stemness and EMT [25].

Signaling networks mediated by microRNAs and EMT-inducing transcription factors tie the EMT process to regulatory networks that maintain stemness can serve as possible targets. For instance, overexpression of miR-125b has been effective in targeting EMT and CSC in HCC by regulating transcription factor SMADs 2 and 4 [72]. Although some miRNAs have been identified to regulate EMT in liver cancer, the role of miRNAs in the EMT and CSC of HCC deserve further investigation [106, 113].

Immunotherapeutic approaches comprise an alternative attractive therapy option, although current studies in targeting CSC and EMT in HCC with immunotherapy are somewhat limited. A recent study showed that EMT signature from multiple cancer types was associated with enrichment of multiple druggable immune targets [114]. Notably, the introduction in clinical practice of agents that target the blockade of immune checkpoints has improved the survival of patients with solid tumors [115, 116]. It would be of interest to examine whether these immune checkpoints are regulated in CSC and EMT phenotypes. For instance, the tumor-restricted pattern of expression of the transcription factor Brachyury and its proven immunogenicity made it an appealing protein for a cancer vaccine strategy targeting EMT [117]. The combination of these therapies with other interventions, such as chemotherapy, radiotherapy, and targeted therapies opens a window of opportunity for the cure of HCC. Thus, next-generation therapies based on increased knowledge of CSC and EMT characteristics and possibly, on the combination of therapeutic interventions, such as immunotherapy and CSC- and EMT-specific-targeted therapies, need to be developed to achieve complete eradications of HCC tumors.

## Conclusions

The notion that CSC and EMT phenotypes play important roles in HCC progression, metastatic competence, therapy resistance, and relapse is a rapidly evolving concept that is contributing to our understanding of HCC pathogenesis and development of effective treatment options for this cancer. Emerging research data indicate that the existence of EMT and CSCs may be related to a high risk for recurrence and poor prognosis for many tumor types. Further investigations into CSC and EMT biology will require additional technological advances for the visualization, isolation, and better characterization of HCC CSCs and EMT phenotype, with robust biomarkers, and elucidation of the signaling pathways that are altered in these tumor cells. Improving our understanding of these cellular states may help to categorize potential targets for novel therapies to preclude relapse. Further research, especially characterization of EMT and CSC phenotypes in HCC is needed for more conclusive information.

As the molecular circuitries underlying EMT and stemness appear closely intertwined, it will be vital to further delineate key molecular players that link these two cellular states. Recent data suggests that CSCs are very plastic and can be associated with both epithelial and mesenchymal states. This plasticity of CSCs further suggests that targeting either state alone may not be sufficient since the CSCs in the alternative state can rapidly regenerate targeted cell populations. Hence, future investigations will be needed to simultaneously target CSCs existing in both epithelial and mesenchymal states to achieve complete tumor eradication. It remains to be seen where combination of conventional chemotherapy with agents that target CSC and EMT states may fundamentally enhance anticancer treatment in HCC.

## Abbreviations

AFP: Alpha-fetoprotein
ABC: ATP-binding cassette
CIC: Cancer-initiating cell
CSC: Cancer stem cell
EMT: Epithelial-to-mesenchymal transition
EGFR: Epidermal growth factor receptor
5-FU: 5-Fluorouracil
FZD2: Frizzled2
HCC: Hepatocellular carcinoma
HCV: Hepatitis C virus
HGF: Hepatocyte growth factor
HIF-1α: Hypoxia-inducible factor-1α
MET: Mesenchymal-to-epithelial transformation
miRNA: Micro RNA
MRP5: Multidrug resistant protein 5
RFA: Radiofrequency ablation
SOX9: Sex-determining region Y-box 9
SP: Side population
Stat3: Signal transducer and activator of transcription 3
SYY: Songyou Yin
TGF: Transforming growth factor
TAM: Tumor infiltrating macrophage
